# miRNA-133 augments coelomocyte phagocytosis in bacteria-challenged *Apostichopus japonicus* via targeting the TLR component of *IRAK-1 in vitro* and *in vivo*

**DOI:** 10.1038/srep12608

**Published:** 2015-07-30

**Authors:** Meng Lu, Peng-Juan Zhang, Cheng-Hua Li, Zhi-Meng Lv, Wei-Wei Zhang, Chun-Hua Jin

**Affiliations:** 1 School of Marine Sciences, Ningbo University, Ningbo, Zhejiang Province 315211, P.R China

## Abstract

In this study, we explored the potential roles of miRNA-133 in regulating TLR pathways in the sea cucumber *Apostichopus japonicus*. Target screening of RNA-Seq data successfully identified interleukin-1 receptor-associated kinase (*AjIRAK−1*) as a putative target of miR-133. This result was further validated by negative expression profiles in *Vibrio splendidus-*challenged coelomocytes and lipopolysaccharide (LPS)-exposed cell cultures. HEK-293T cells transfected with a dual-luciferase reporter fused to the 3′UTR of wild-type or mutant *AjIRAK-1* exhibited a 52.9% reduction in luciferase activity (p < 0.01) compared to controls. Co-infection with a miR-133 mimics or a specific siRNA targeting *AjIRAK-1* significantly repressed the mRNA and protein expression levels of *AjIRAK-1* and its downstream molecules, such as *AjTRAF6* and *Ajp105,* in primary coelomocytes. In contrast, a miR-133 inhibitor significantly increased the expression of these TLR pathway members. The injection of miR-133 agomir or *AjIRAK-1* siRNA into sea cucumbers not only decreased the expression of *AjIRAK-1* and its downstream molecules but also significantly increased *V. splendidus* coelomocyte phagocytosis. All of the present data provide direct evidence that miR-133 is involved in TLR cascade modulation through *AjIRAK-1* targeting to promote *V. splendidus* coelomocyte phagocytosis in these non-model invertebrates.

The innate immune system defends against a spectrum of microbial pathogens that, in terms of environmental prevalence, range from common to rare. Host organisms respond to an infection by initiating both inflammatory and immune responses in an attempt to clear pathogens from their systems. Phagocytosis is the first line of this host-pathogen interaction, and it is tightly controlled by pattern recognition receptors (PRRs). Three categories of PRRs are considered to be engaged in this process, and the intimate links between TLR and phagocytosis have been fully elucidated in vertebrates^1^. Toll-like receptors are a family of conserved type I transmembrane proteins that function as pattern recognition receptors for lipopolysaccharides (LPS) and other pathogen-associated molecular patterns in most model organisms, such as fruit flies and humans^2^. TLR signaling pathway activation is hierarchical, and MyD88 recruits IRAK-4 and IRAK-1 in succession^3^; phosphorylated IRAK-1 then mediates the recruitment of TRAF6. Following its dissociation from the receptor, the IRAK4-IRAK1-TRAF6 complex phosphorylates transforming growth factor-β (TGF-β)–activated kinase 1 (TAK1), TGF-β activated protein kinase 1-binding protein 1 (TAB1), and TGF-β-activated protein kinase 1-binding protein 2 (TAB2)^4^. TAK1 phosphorylation leads to IKK activation, causing IκB degradation, NF-κB activation and transcription of proinflammatory cytokines^3^,^4^. In recent years, TLR-like molecules and their downstream targets have been identified and characterized in some invertebrates, such as shrimp^5^,^6^, scallops^7^,^8^ and sea cucumbers^9^,^10^, suggesting the existence of TLR pathway-mediated innate immunity in these lower marine animals.

microRNAs (miRNAs) are highly conserved, small non-coding RNAs that regulate gene expression by binding to the 3′-untranslated regions (UTRs) of target genes, typically resulting in protein translation repression or mRNA cleavage^11^. To date, more than 30,000 miRNAs have been identified in at least 206 species^12^. As one of the most abundant classes of gene regulators, an increasing number of miRNAs have been found to play key roles in both innate and adaptive immune responses in reaction to a pathogenic challenge^13^. He *et al*. reviewed approximately 20 miRNAs that mediate an immune response via Toll-like receptor signaling pathway regulation in vertebrates, including miR-146a, miR-155, miR-21 and miR-301a^12^; the validated targets included *TRAF6*, *IRAK-1*, *IKK* and *NF-κB*^14^. We speculated that additional miRNAs may also participate in the immune response and may potentially impact the outcome of the host-pathogen interaction. Moreover, the detailed mechanisms by which miRNAs regulate host immunity and the inflammatory response in aquatic organisms are largely unclear.

The sea cucumber (*Apostichopus japonicus*) is one of the most important aquaculture species in China. However, various diseases caused by bacteria and protozoa often occur in cultured *A. japonicus* populations following the development of intensive cultures. This can result in environmental deterioration that causes enormous losses of the aquaculture species and is currently one of the limiting factors in the sustainable development of this industry^15^,^16^. Among these diseases is skin ulceration syndrome (SUS), a major issue in sea cucumber cultivation that leads to massive death during the larval period. Researchers focusing on the pathogens responsible for SUS have identified multiple pathogens, including aspherical virus^17^, *Vibrio splendidus* and *Pseudomonas* spp. It is widely accepted that *V. splendidus* is the primary pathogen responsible for SUS outbreaks in cultured *A. japonicus* populations^18^,^19^. To establish a highly effective disease control strategy, different members of the TLR pathway have been identified in sea cucumbers, such as Toll^10^, Tollip, IκB^20^, MyD88 and TRAF6^21^, and expression analyses have further supported their potential roles in the pathogen-mediated response.

To fully address how these molecules are activated and modulated during this host-pathogen interaction, two miRNA libraries were sequenced via deep sequencing, and several key miRNAs were identified^22^. Among them, miR-133 displayed significant upregulation in diseased *A. japonicus*^22^. Recent emerging evidence indicates miR-133 as a key regulator in cell development^23^, proliferation^24^, and the occurrence of heart disease and cancers^25^,^26^,^27^. For example, miR-133 is significantly overexpressed in the plasma of acute myocardial infarction (AMI) patients compared to non-AMI individuals and improves patient stratification when used as a potential biomarker^28^. In a study of zebrafish heart regeneration, Yin *et al*. found that following injury, high levels of miR-133 inhibited myocardial regeneration and that miR-133 inhibitors promoted cardiomyocyte proliferation^29^. However, the connection between miR-133 involvement in bacterial infections and the activation of its downstream targets has yet to be investigated in both invertebrates and vertebrates.

Given the important roles of the TLR signaling pathway in the innate immune response, we identified miR-133 targets in sea cucumber coelomocytes via RNA sequencing and elucidated its regulatory roles via loss- and gain-of-function analyses *in vitro* and *in vivo*. Moreover, the connections among miR-133, TLR and phagocytosis were also investigated in pathogenically challenged sea cucumber coelomocytes by a colony-forming unit (CFU) assay. All of our results provide new evidence that miR-133 is involved in the anti-bacterial response process in sea cucumbers via NF-κB dysregulation.

## Results

### Prediction of the miR-133 targets

miR-133 displayed significantly different expression profiles between SUS-diseased and healthy sea cucumber coelomocytes (22). To elucidate its function, putative miR-133 targets were calculated and shown in Table 1. A total of 20 genes were predicted to be miR-133 targets based on the miRNA binding site recognized by the miRanda program. The potential targets included several immunity-related genes, such as *AjIRAK-1*, superoxide dismutase, lysozyme, nitric oxide synthase and heat shock protein. Among them, *AjIRAK-1* had the highest probability of being a candidate target based on its precisely matched seed sequence in the 3′UTR and had the highest single-residue pair score and lowest free energy.

### cDNA cloning and sequence analysis of *AjIRAK-1*

**Figure 1:**
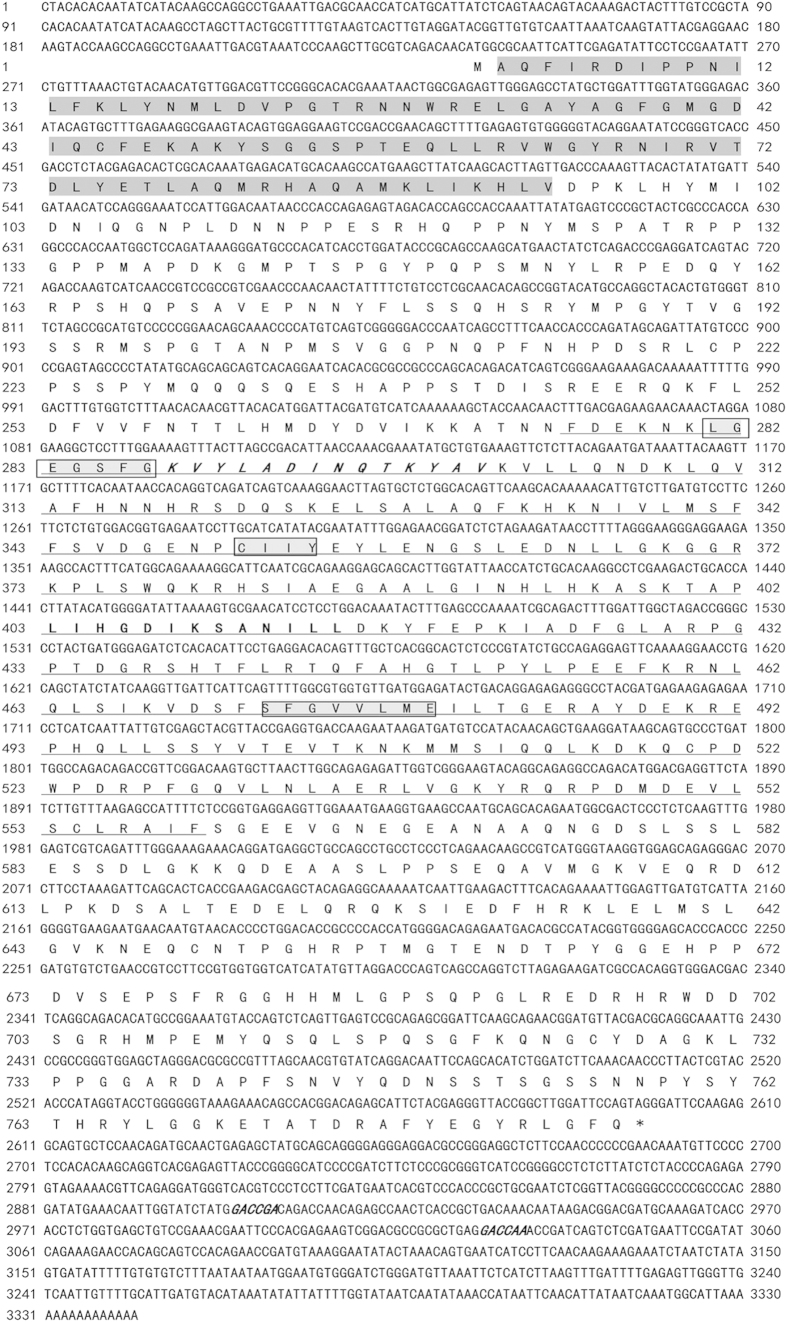
The complete cDNA sequence of interleukin-1 receptor-associated kinase 1 from *Apostichopus japonicus* and its predicted amino acid sequence. A typical death domain (DD) and a kinase domain are shadowed and underlined, respectively. Three highly conserved motifs are shown boxed. The ATP-binding site is italicized and bolded. The kinase-action site is bolded. The two putative biding sites of miR-133 are inlicited and bolded.

To further characterize the putative miR-133 binding sites, full-length *AjIRAK-1* cDNA was cloned; the sequence was deposited in the GenBank database under accession number KJ918751 (Fig. 1). *AjIRAK-1* is 3342-bp long with a 234-bp 5′UTR and a 744-bp 3′UTR containing two perfect miR-133 binding sites, as predicted by the miRanda program (Fig. 1 italicied, bolded and underlined). A putative ORF of 2364 bp encodes 787 amino acids, with a predicted MW of 88.46 kDa and a theoretical pI of 6.47. SMART analysis revealed that the predicted amino acid sequence of *AjIRAK-1* contains a conserved death domain (DD) (2 aa-94 aa) and a central kinase domain (275 aa-559 aa) with ATP-binding and serine/threonine protein kinase-activating motifs. Three highly conserved sites (LGEGSFG, CIIY, and SFGVVLME) in the kinase domain were also identified.

**Figure 2:**
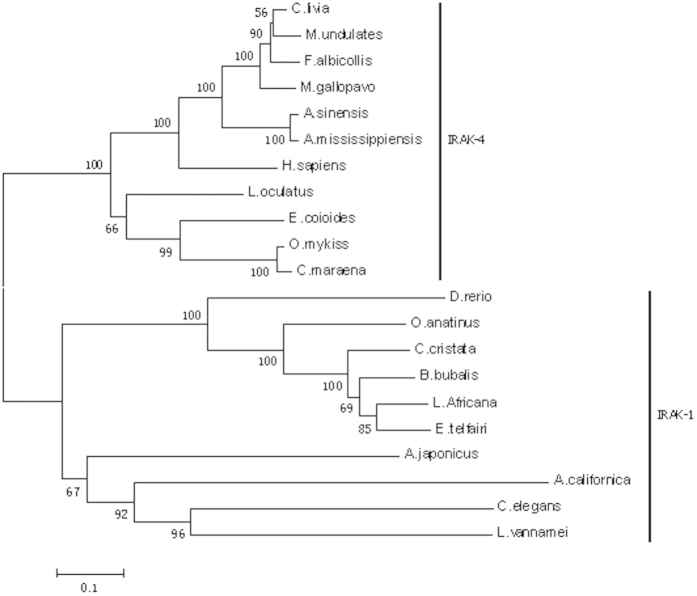
The phylogenetic tree constructed based on the IRAK amino acid sequence using the neighbor-joining algorithm. The GenBank accession numbers used are as follows:*Ornithorhynchus anatinus* IRAK-1 XP_007667869; *Amphimedon queenslandica* IRAK-4 XP_003388757; *Lepisosteus oculatus* IRAK-4 XP_006642971; *Aplysia californica* IRAK-1 XP_005095880; *Caenorhabditis elegans* IRAK-1 NP_001255742; *Aplysia californica* IRAK-1 XP_005095879; *Bubalus bubalis* IRAK-1 XP_006043774; *Salmo salar* IRAK-4 NP_001135238; *Meleagris gallopavo* IRAK-4 XP_003202068; *Columba livia* IRAK-4 XP_005499983; *Epinephelus coioides* IRAK-4 AGQ48127; *Litopenaeus vannamei* IRAK-1 AGU41814; *Oncorhynchus mykiss* IRAK-4 CBI63176; *Melopsittacus undulates* IRAK-4 XP_005148151; *Alligator sinensis* IRAK-4 XP_006015992; *Loxodonta Africana* IRAK-1 XP_003421767; *Danio rerio* IRAK-1 XP_005166760; *Condylura cristata* IRAK-1 XP_004695245; *Ficedula albicollis* IRAK-4 XP_005039421; *Echinops telfairi* IRAK-1 XP_004717621; *Alligator mississippiensis* IRAK-4 XP_006272300; *Bos mutus* IRAK-1 ELR46602; *Coregonus maraena* IRAK-4 CBI63179; *Pan paniscus* IRAK-4 XP_003825792; *Homo sapiens* IRAK-4 AAM15772.

To determine the evolutionary position of AjIRAK-1 among the other corresponding family members, a phylogenetic tree was constructed using the neighbor-joining method (Fig. 2). The results showed that IRAK-1 and IRAK-4 are found in two different clades, each populated by vertebrates and invertebrates. AjIRAK-1 forms a branch with IRAK-1, which originates from *Caenorhabditis elegans*, *Litopenaeus vannamei* and *Aplysia californica*.

### Expression analysis of *AjIRAK-1* and miR-133 *in vitro* and *in vivo*

**Figure 3:**
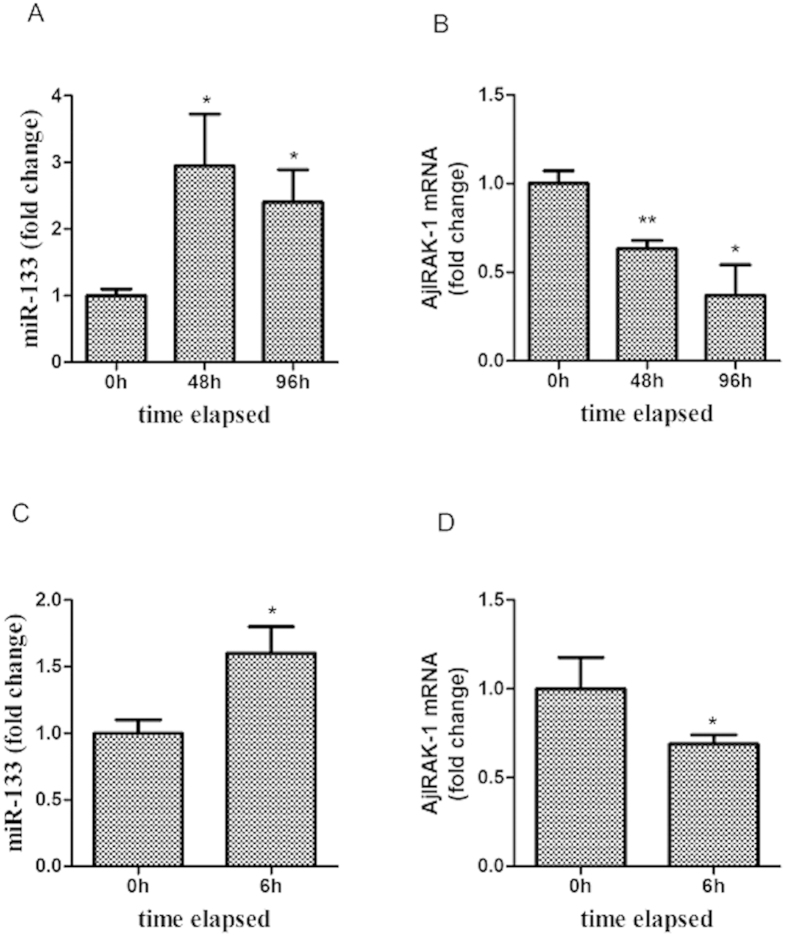
The time-course expression patterns of miR-133 and AjIRAK-1 in *V. splendidus*-challenged *A. japonicus* (**A**,**B**)—and LPS-exposed primary cultured cells (**C**,**D**) as measured by qRT-PCR. Data are expressed as the mean ± SD (n = 3).

The expression profiles of miR-133 and *AjIRAK-1* in *V. splendidus-*challenged sea cucumbers and LPS-treated cultured coelomocytes are shown in Fig. 3. The levels of miR-133 were significantly increased in both the pathogen-challenged sea cucumbers and the LPS-exposed primary coelomocytes, with approximately 3.0-fold and 1.5-fold greater expression, respectively, compared to the controls (Fig. 3A,C). In contrast, the *AjIRAK-1* mRNA transcripts displayed a sharp reduction at all of the examined time points. The lowest expression levels were detected at 96 h, with a 0.37-fold decrease compared to that of the controls for the *in vivo* assay, and at 6 h, with a 0.69-fold decrease for the *in vitro* assay (Fig. 3B,D).

### Luciferase reporter assay

**Figure 4:**
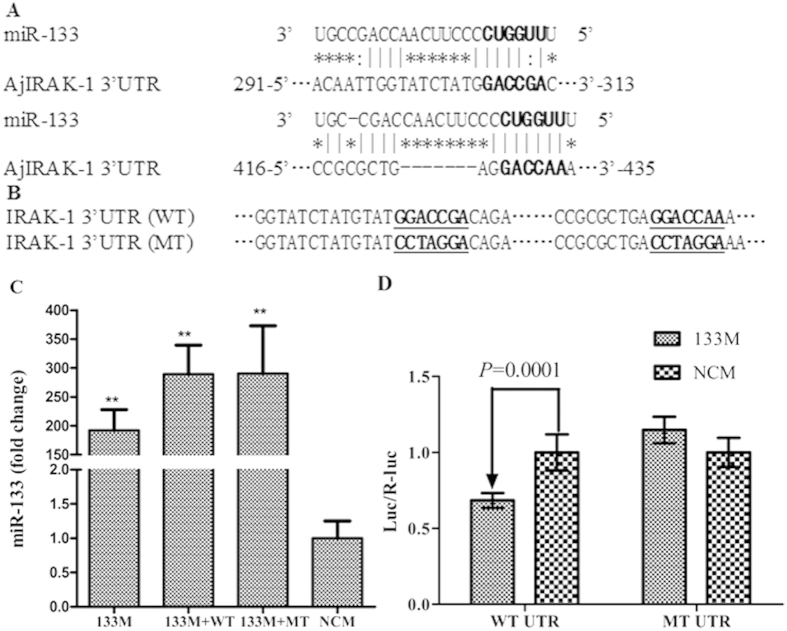
A schematic of the luciferase reporter assay used to validate the interaction between miR-133 and the 3′UTR of AjIRAK-1. The bold font indicates the “seed” regions. The AjIRAK-1 3′UTRs of the wild type and full mutants are shown in A and B. **A**: Two predicted miR-133 binding sites in the AjIRAK-1 3′UTR. **B**: Mutant sequences of the AjIRAK-1 3′UTR seed sequence. WT: Wild-type; MT: Mutant type. **C**:The relative expression levels of miR-133 after miR-133 mimics and plasmid coinfection. NCM: Negative control of miR-133 mimics; 133M: miR-133 mimics; 133M + WT: miR-133 mimics and wild-type plasmid coinfection; 133M + MT: miR-133 mimics and mutant-type plasmid coinfection; D: Normalized luciferase activity of a reporter containing the WT or MT reporter constructs of AjIRAK-1 in HEK293T cells co-transfected with negative control of miR-133 mimics (NCM) or miR-133 mimics (133M). The data are represented as the mean ± SD (n = 6).

The results of the luciferase assay, as well as the miR-133 mutant information, are shown in Fig. 4. Following the transient co-transfection of HEK-239T cells with pMIR-REPORT construct, pRL-CMV and miR-133 mimics, we observed a 289.58-fold inceases for wild-type (p = 0.001) and 290.58-fold increase for mutant type (p = 0.005) in miR-133 expression level compared with each control groups, respectively (Fig. 4C). The activity of the luciferase construct containing the entire 3′UTR of *AjIRAK-1* was suppressed by approximately 52.9% (P < 0.01) when paired with ectopic miR-133 expression (Fig. 4D). The suppressed luciferase activity was abolished when a 3′UTR mutant was introduced into the recombinant plasmid (Fig. 4D).

### Loss- and gain-of-function analyses of miR-133 *in vitro*

**Figure 5:**
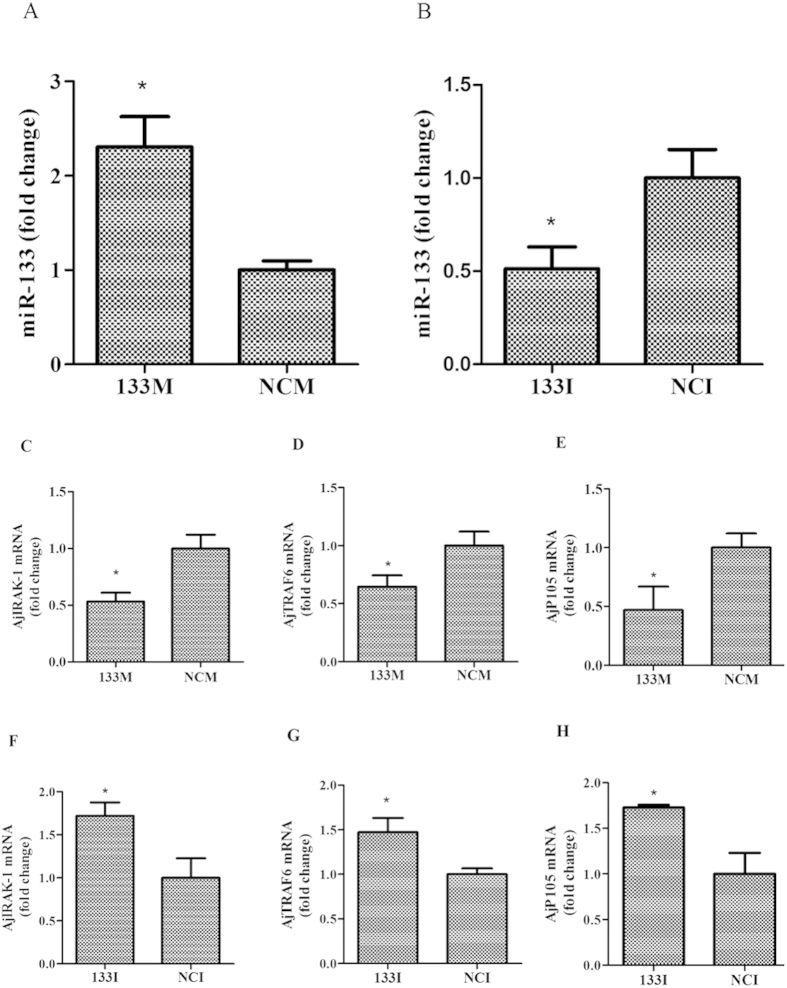
The expression profiles of miR-133, AjIRAK-1 and the downstream signaling molecules after transfection with miR-133 inhibitors (**B**,**G**–**J**) or mimics (**A**,**C**–**F**) in primary cultured coelomocytes. Three biological replicates were performed in the experiment and the obtained data are expressed as the mean ± SD (n = 3). NCM: miR-133 mimics control; NCI: miR-133 inhibitor control; 133M: miR-133 mimics; 133I: miR-133 inhibitor.

**Figure 6:**
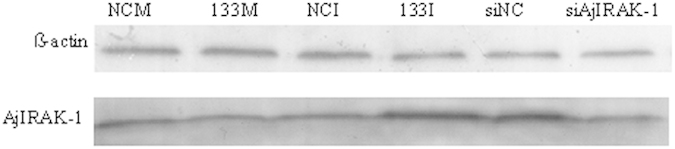
Western blot analysis of AjIRAK-1 protein expression after miR-133 abberrant expression and *AjIRAK-1* interference in primary coelomocytes. NCM: Negative control of miR-133 mimics; 133M: miR-133 mimics; NCI: Negative control for the miR-133 inhibitor; 133I: miR-133 inhibitor; siNC: Negative control for the *AjIRAK-1* siRNA; siAjIRAK-1: *AjIRAK-1* interference.

To address the connection between miR-133 and the TLR signaling cascades in sea cucumbers, the expression profiles of *AjIRAK-1*, *AjTRAF6*, *Ajp105* and were examined by loss- and gain-of-function analyses of miR-133 in primary cultured coelomocytes (Fig. 5). miR-133 overexpression significantly decreased the expression of *AjIRAK-1*, *AjTRAF6*, and *Ajp105* by 0.53-fold, 0.64-fold, and 0.46-fold, respectively, compared to that of the control group (Fig. 5C–E), whereas miR-133 inhibitors elevated the expression of the molecules by 1.72-fold, 1.47-fold, 1.73-fold, respectively, compared with the expression of the control group (Fig. 5G–I). Consistently, western blot analysis of miR-133 aberrant expression revealed that the protein abundance of AjIRAK-1 was decreased upon miR-133 over-expression and increased upon miR-133 inhibition (Fig. 6).

### siRNA interference with *AjIRAK-1* in primary coelomocytes

**Figure 7:**
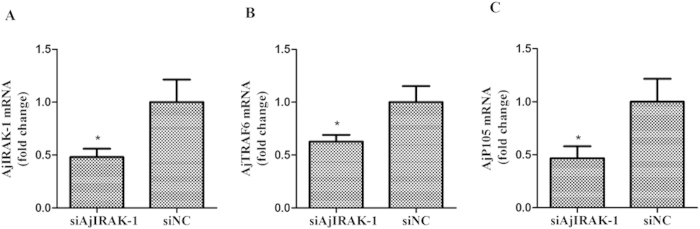
The mRNA expression levels of *AjIRAK-1* and its downstream molecules after AjIRAK-1 interference using siRNA. Three biological replicates were performed in the experiment and the obtained data are expressed as the mean ± SD (n = 3). siAjIRAK-1: AjIRAK-1 siRNA transfection; siNC: control siRNA transfection.

The expression patterns of *AjIRAK-1* and its downstream signaling molecules were further analyzed in an *AjIRAK-1* knockdown experiment (Fig. 7). *AjIRAK-1* levels were significantly reduced by 0.48-fold at mRNA level (Fig. 7A) and by 2.35-fold in protein abundance (Fig. 6) after siRNA transfection in the primary coelomocytes. Similar downregulation trends were also detected for other downstream molecules, such as a 0.63-fold decrease in *AjTRAF6* (p < 0.01) and a 0.47-fold decrease in *Ajp105* (p < 0.01) (Fig. 7B,C).

### Overexpression of miR-133 and interference with *AjIRAK-1*-augmented *V. splendidus* phagocytosis *in vivo*

**Figure 8:**
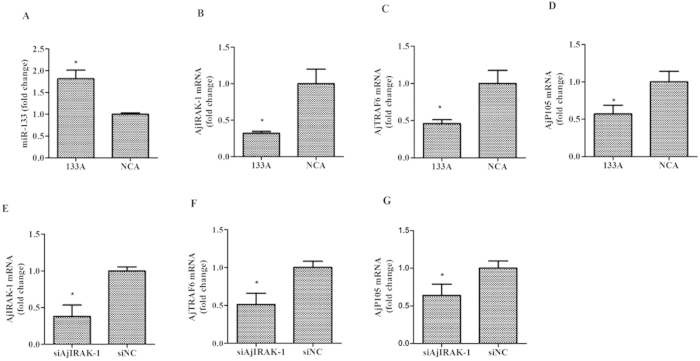
The mRNA expression levels of *AjIRAK-1* and its downstream molecules after miR-133 agomir or AjIRAK-1 interference *in vivo*. Three biological replicates were performed in the experiment and the obtained data are expressed as the mean ± SD (n = 3). 133A: miR-133 agomir; NCA: Negative control of miR-133 agomir;siAjIRAK-1: AjIRAK-1 siRNA transfection; siNC: control siRNA transfection.

**Figure 9:**
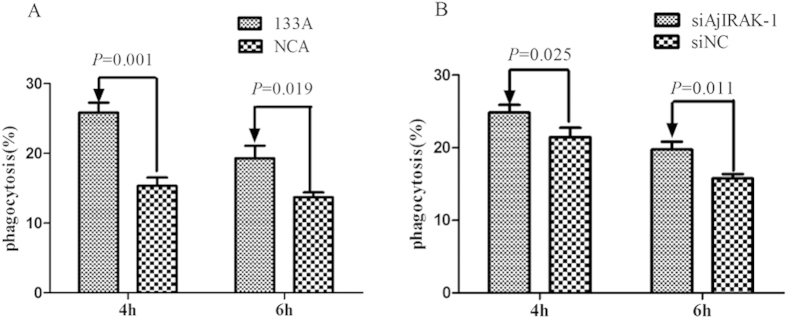
Coelomocyte phagocytosis activity after miR-133 agomir or AjIRAK-1 siRNA injection *in vivo* by the CFU assay. Three biological replicates were performed in the experiment and the obtained data are expressed as the mean ± SD (n = 3).

The injection of miR-133 agomir or *AjIRAK-1* siRNA into sea cucumbers clearly inhibited *AjIRAK-1* expression levels and its downstream molecules (Fig. 8). We then compared the *V. splendidus* phagocytosis ability of sea cucumber coelomocytes in the presence of miR-133 agomir or *AjIRAK-1* siRNA. As shown in Fig. 9A, miR-133 overexpression led to a nearly 10% increase in intracellular *V. splendidus* at 4 h and 6.6% at 6 h compared with its control group. *AjIRAK-*1 siRNA treatment also increased the internalization of *V. splendidus* by 3.4% at 4 h and 2.7% at 6 h compared with that in the control group (Fig. 9B).

## Discussion

Phagocytosis, and the subsequent degradation of pathogens, has been demonstrated to play an essential role in the host immune response toward bacterial infection^30^. Several types of immune cells are able to engulf and kill invasive bacteria, including macrophages and dendritic cells in vertebrates and hemocytes or coelomocytes in invertebrates^31^. However, the detailed mechanisms responsible for phagocytosis regulation are largely unknown in invertebrates, such as sea cucumbers. Here, we report a novel role for miR-133 in promoting pathogen phagocytosis in sea cucumber coelomocytes through IRAK-1 targeting, an important component of TLR cascades, *in vivo* and *in vitro*. This finding provides a better understanding of the host anti-bacterial response in this non-model invertebrate.

There is growing evidence that indicates that TLR induction of NF-κB activation is involved in the regulation of the phagocytosis process in macrophages^31^, with most studies revealing positive phagocytosis regulation by TLR. Blander and Medzhitov^32^ indicated that MyD88-deficient macrophages had a lower capacity to internalize *S. aureus* than wild-type cells. TLR4 was also found to accelerate bacterial phagocytosis in human enterocytes^33^. Surprisingly, we found that phagocytosis activity was significantly elevated at all of the examined time points by a CFU assay, including after miR-133 agomir injection and *AjIRAK-1* siRNA transfection (Fig. 7). TLR silencing by miR-133 increased the live bacterial number from 1.55 × 10^5^ CFU (control group) to 2.59 × 10^5^ CFU 4-h post-infection. Consistently, the values changed from 2.14 × 10^5^ CFU to 2.48 × 10^5^ CFU in *AjIRAK-1* knockdown experiments. The enhanced phagocytosis might be a “danger signal” to induce humoral immunity. Moretti and Blander^1^ showed that phagocytosis is a necessary precedent to activate cytosolic PRRs and to assemble canonical and non-canonical inflammasomes, which leads to strong pro-inflammatory responses that combat pathogen entry. However, reduced IRAK-1 expression is reportedly responsible for bacteria-induced tolerance, a mechanism that serves to mitigate excessive and potentially harmful inflammatory reactions^34^. Studies of airway epithelial cells have indicated that the decreased IRAK-1 protein content and kinase activity inhibit *Pseudomonas aeruginosa*-stimulated NF-κB transcriptional activity and mediate the adaptation of epithelial cells and tolerance to *P. aeruginosa*^35^. Furthermore, studies of the LPS-tolerant phenotype have also demonstrated that reduced IRAK-1 expression and impaired TLR4-MyD88 complex formation are associated with bacterial lipoprotein-induced self-tolerance and cross-tolerance to LPS^36^,^37^.

Numerous investigations have supported the role of miRNAs in the immune response and in NF-κB regulation^17^,^38^,^39^; more than 20 miRNAs have been shown to participate in TLR signaling pathway regulation, such as miR-146a, miR-155, miR-148 and miR-21^12^. miR-146 was first reported to negatively regulate the TLR and RIG-I signaling pathways via the 3′UTR of the *IRAK-1* and *TRAF6* genes^40^,^41^. Additionally, miR-21 has been implicated in the control of the inflammatory response by targeting *IRAK-1* and by acting as a molecular switch between the pro-inflammatory (IRF) and anti-inflammatory (IFN-α) states in hepatitis C virus (HCV)-infected cells^42^. Here, we describe the regulation of TLR signaling cascades by miR-133 via *IRAK-1* targeting in *A. japonicus* using multiple molecular approaches. The binding sites for miR-133 were confirmed via a luciferase assay combined with mutant analysis. Co-infection with a miR-133 inhibitor significantly increased the expression of *AjIRAK-1* and its downstream molecules, such as *AjTRAF6* and *Ajp105*, in primary coelomocytes. We also screened all of the candidate genes in the TLR cascade, revealing *AjIRAK-1* to be a putative target for miR-133; this excludes the possibility that miR-133 directly modulates the gene expression of downstream members. After *AjIRAK-1* is induced by an miR-133 inhibitor, the feed-back regulation of TLR cascade activation would increase the other downstream molecules’ expression through other regulatory processes. Consistently, miR-133 mimics or a siRNA injection also inhibited the activation of TLR signal-related molecules *in vivo* and significantly increased the survival of invasive bacteria.

The miR-133 family has been rigorously investigated as one of the key essential factors in human cancers^26^, and recent studies have demonstrated that the majority of the validated targets displayed an indirect or direct connection with the TLR cascade. In cardiomyocytes, miR-133 suppresses cell apoptosis via *Hsp70* targeting^43^; this molecule was demonstrated to participate in TLR signaling pathway activation via TLR2 and TLR4 stimulation^44^. Glutathione S-transferase P1 (GSTP1) was also shown to be directly regulated by miR-133 in head and neck squamous cell carcinoma and bladder cancer cell lines^45^,^46^. In turn, GSTP1 was found to be involved in tumor necrosis factor-α (TNF-α)-triggered signaling activation through an interaction with TRAF2 homology molecules in TLR cascades^47^. CXC chemokine receptor 4 (CXCR4) and transcription factor Sp1 are two other well-documented targets of miR-133 in humans^48^,^49^. Current evidence indicates that CXCR4 is connected to pro-inflammatory (NF-κB) responses through its control of G protein signaling and the PI3K-Akt signaling pathway. However, Sp1 has been shown to act as a critical component of the DNA demethylation-dependent upregulation of TLR2 expression in cystic fibrosis (CF) epithelial cells^50^. All of these reports collectively support the connection between miR-133 and the TLR pathway.

As a group of key regulators of gene expression, miRNAs are considered to be promising targets for cancer therapy in humans. Recent advances involving the use of miRNA-based technologies may provide alternative approaches for the stable silencing of target genes in several diseases, such as miR-483-3p and miR-1202 in patients with a left ventricular assist device^51^, miR-630 in colorectal cancer^52^, and miR-320, miR-320b and miR-629 in progressive multifocal leukoencephalopathy^53^. To achieve the goal of controlling SUS outbreaks in sea cucumbers, we suggest the use of miR-133 as a potential biomarker and therapeutic target for *V. splendidus*-challenged sea cucumbers.

## Methods

### Prediction of the miR-133 targets

The miR-133 targets were predicted via using the miRanda v3.01 toolbox by screening our previous transcriptome data^54^,^55^. All of the mRNAs used for target prediction came from the differentially expressed unigenes obtained above, where the candidate targets with scores less than the threshold parameter of S > 90 (single-residue pair scores) and a minimum free energy lower than −17 kcal/mol were selected for binding site analysis. The promising candidate with the highest score and lowest free energy was selected for further analysis.

### Experimental animals and conditions

Eighty healthy adult sea cucumbers (*A. japonicus*, 120 ± 15 g) were obtained from Bowang Aquaculture Company in April 2014 and were acclimatized in aerated natural seawater (salinity, 28 psu; temperature, 16 °C) for three days. Pathogenic microorganism *V. splendidus* was initially isolated from SUS-diseased sea cucumbers and kept in our lab. The bacteria were inoculated in liquid 2216E broth (Tryptone, 5 g L^−1^; yeast extract, 1 g L^−1^; pH 7.6) and cultured at 28 °C and 220 rpm overnight. The cultures were centrifuged at 5,000 × *g* for 5 min to harvest the bacteria and then re-suspended in filtered seawater. The clonal forming unit was determined by a serial dilution assay. For the challenge experiments, sea cucumbers were randomly divided into four tanks with each containing 20 individuals. The two experimental groups were infected with live *V. splendidus* at a final concentration of 10^7^CFU mL^−1^. The other two tanks served as control groups. Coelomic fluids were collected at 0-, 48- and 96-h post infection. The samples were then stored at −80 °C for RNA extraction and cDNA synthesis.

### Cloning of the full-length IRAK-1 cDNA from *A. japonicus* (*AjIRAK-1*)

The partial *IRAK-1* cDNA sequence was extracted from our completed *A. japonicus* transcriptome data^55^. BLASTx analysis of the fragment revealed that the sequence contained the complete 5' end compared to that of other reported counterparts. No polyA tail was detected in the *AjIRAK-1* fragment. Therefore, gene specific primers (Table 2) for a 3′RACE experiment were designed to obtain the full-length *AjIRAK-1* cDNA. The desired PCR products were separated by agarose gel electrophoresis, purified using a gel extraction kit (OMEGA, USA), and ligated into the pMD18-T vector (TaKaRa, Japan). The recombinant plasmids were then transformed into competent *Escherichia coli* DH5α cells and were used for positive clone screening by PCR analysis. Three clones were bi-directionally sequenced at Sangon Biotech (Shanghai, China).

### Sequence analysis of the AjIRAK-1 cDNA

The *AjIRAK-1* cDNA sequences were analyzed using the BLAST algorithm at the National Center for Biotechnology Information (http://www.ncbi.nlm.nih.gov/blast), and the predicted amino acid sequence was analyzed using the Expert Protein Analysis System (http://www.expasy.org/). A phylogenetic tree was constructed based on the full-length amino acid sequences of the different original IRAKs using the neighbor-joining algorithm in the MEGA6 software package.

### 3′UTR luciferase reporter assays

The complete 3′UTR of wild-type *AjIRAK-1* was amplified by gene-specific primers with restricted endo-enzyme sites. Two putative miRNA binding sites were mutated using a PCR approach and served as the mutant type of *AjIRAK-1*. All purified fragments were digested by double enzymes and ligated into the digested pMIR-REPORT vector. These clones were further confirmed by sequencing before the luciferase reporter assay. For the transfection experiment, HEK-293T cells were seeded into a 96-well white TC plate for a 100-μL total volume. Two solutions were prepared in each well as follows: the first solution contained 0.2 μg of pMIR-REPORT constructs containing either the wild-type or mutated *AjIRAK-1* 3′UTR and and 0.01 μg of pRL-CMV with 0.25 μL of transfection reagent. The second solution was miR-133 with 100 nM miR-133 mimics and 0.25 μL of transfection reagent. Twenty-five microliters of each solution were mixed together and incubated at room temperature for 20 min, then the solutions were replaced by 50 μL of medium in each well. Forty-eight hours post-transfection, the cells were collected for activity determination using the Dual-Luciferase Reporter Assay System (E1910, Promega). The efficiency of miR-133 transfection was confirmed by real-time quantitative PCR with RNU6B as internal control. The luciferase signal was calculated based on the luciferase signal ratio between the two constructs, pMIR-REPORT and pRL-CMV, which could be used to normalize the protein content among different samples. All of the experiments were performed in six replicates.

### Cell culture and LPS exposure

Sea cucumbers were sterilized in 7% benzalkonium bromide and 75% ethanol for approximately 2 min. Subsequently, the sea cucumbers were dissected using an aseptic surgery technique as previously described^56^. The coelomic fluids were collected and mixed with an equal volume of anticoagulant solution (0.02 M EGTA, 0.48 M NaCl, 0.019 M KCl, and 0.068 M Tris-HCl, pH 7.6). The cell suspension was filtered through a 100-μm nylon mesh to remove large tissue debris and centrifuged at 800 x *g* for 10 min at 16 °C. The cells were washed twice with isotonic buffer (0.001 M EGTA, 0.53 M NaCl, and 0.01 M Tris-HCl, pH 7.6) and re-suspended in Leibovitz’s L-15 cell culture medium (Invitrogen, USA) supplemented with penicillin (100 U mL-1), streptomycin sulfate (100 μg mL-1), and NaCl (0.39 M) to adjust the osmotic pressure. Next, 1000-μL aliquots of cell suspension were dispensed into 24-well microplates and cultured for 6 h at 16 °C in a black room. For the LPS exposure, the primary cultured coelomocytes were exposed to 1 mg mL^−1^ of LPS for 0 h and 6 h. Following the LPS exposure, the cells were washed in PBS, centrifuged at 800 × *g* and 4 °C for 5 min, and stored at –80 °C for the subsequent expression analysis.

### Transfection of the miRNA-133 mimics and inhibitor

The miR-133 mimics and inhibitors were synthesized at GenePharma (Shanghai, China) and were shown in Table 2. For miR-133 mimics and inhibitor transfections, HiPerFect transfection reagents (Qiagen, Germany) were used for the transfection experiment. The miR-133 mimics or inhibitor (2.0 μL of 20 μM), as well as each negative control, were mixed with an equal volume of HiPerFect transfection reagents. The mix was then transfected into 1000 μL of primary cultured cells. Twenty-four hours post-transfection, the cells were harvested and washed with cold PBS, then centrifuged at 800 × *g* and 4 °C for 5 min in preparation for the subsequent expression analysis.

### *AjIRAK-1* silencing

Small interfering RNA (siRNA) targeting *AjIRAK-1* and a negative control were designed and synthesized by GenePharma (Shanghai, China). The detailed sequence information is shown in Table 2. Specific siRNA oligonucleotides (2.0 μL of 20 μM) or the negative control were mixed with 2.0 μL of siRNA-specific transfection reagent (GenePharma, Shanghai). Then, the mix was added to 1000 μL of primary cultured cells for 24 h. The treated and control coelomocytes were collected for expression analysis.

### Western blot analysis

The generation of reconmbinant proteins and preparation of antiserum of IRAK-1 and β-actin were conducted to our previous work^57^. For the western blot assay, the samples were washed twice in ice-cold PBS, and the protein was extracted using the Total Protein Extraction Kit (Sangon, China) according to the manufacturer’s instructions. Subsequently, the protein concentration was measured using the BCA Protein Assay Kit (Sangon, China). For SDS-PAGE, 25 μg of protein from each sample was used, followed by electrophoretic transfer to a 0.45-mm pore nitrocellulose membrane using an ECL Semi-dry Blotter (Amersham Biosciences). After blocking with 5% skimmed milk (in 20 mM Tris-HCl, 150 mM NaCl, and 0.05% Tween-20) at 37 °C for 1 h, the membranes were incubated with IRAK-1 or β-actin polyclonal antibodies diluted 1:500 in 5% skimmed milk at room temperature for 2 h. The membranes were washed three times with TBST (20 mM Tris-HCl, 150 mM NaCl, and 0.05% Tween-20) and subsequently incubated with goat-anti-rat IgG (Beyotime) diluted 1:1000 in 5% skimmed milk at room temperature for 1.5 h. After washing three times with TBST for 10 min each, the membrane was incubated in Western Lightning-ECL substrate (Perkin Elmer) prior to exposure onto X-OMAT AR X-ray film (Eastman Kodak, Rochester, NY). The protein bands were quantified by using the BioRad Quantity one software package, and the results were derived from the statistical analysis of three independent experiments.

### miR-133 gain-of-function assay in sea cucumbers *in vivo*

The miR-133 agomir and the agomir control were designed for the *in vivo* assay and synthesized by GenePharma (Shanghai, China). The sequence information is shown in Table 2. These agomirs were then dissolved into RNase-free water to obtain a working solution of 20 μM. We mixed 10 μL of each agomir or siRNA with 10 μL of transfection reagent and 80 μL of PBS to serve as the transfection solution. Twenty sea cucumbers (60 ± 10 g) were infected with 100 μL of miR-133 agomir, the agomir control, siIRAK1 or the siRNA negative control. Twenty-four hours later, the treated and control coelomocytes were collected for expression analysis. The assays described above were biologically repeated three times.

### RNA isolation and real-time quantitative PCR

Total RNA was isolated using RNAiso Plus (TaKaRa), and the cDNA was synthesized using a PrimeScript miRNA RT-PCR Kit (TaKaRa). A real-time quantitative PCR experiment, using SYBR Green detection chemistry (TaKaRa), was performed on a Rotor-Gene 6000 real-time quantitative PCR detection system. The primer information for the real-time quantitative PCR is shown in Table 2. RNU6B and 18S rRNA served as internal controls to normalize the miRNA or the targets for quantification, respectively. Each reaction was performed in a final volume of 20 μL, which contained 2 μL of the cDNA, 1 μL of each primer (10 μM), 6 μL of RNase-free water and 10 μL of the SYBR Green PCR Master Mix (TaKara). The amplification profile was as follows: denaturation at 94 °C for 5 min, followed by 40 cycles of 94 °C for 15 s, 60 °C for 30 s and 70 °C for 30 s. At the end of the PCR cycles, melting curve analyses were performed. The Ct value is defined as the fractional cycle number at which the fluorescence passes the fixed threshold. Each sample was analyzed in triplicate.

### Colony-forming unit (CFU) assay

The transfection experiments for miR-133 agomir, the agomir control, siIRAK1 or the siRNA negative control were conducted according to the description in “miR-133 gain-of-function assay in sea cucumbers *in vivo.”* After 24 h, 100 μL of live *V. splendidus* (10^7^ CFU mL^−1^) was injected into each sea cucumber. Four or six hours later, the coelomic fluid was collected and incubated with gentamicin (100 μg mL^−1^, Sigma) for 2 h to kill the extracellular bacteria. The cells were washed three times with cold PBS and then lysed in 1 mL of distilled water. Quantitative culturing was performed using 10-fold serial dilutions. Aliquots of each dilution were inoculated in triplicate onto 2216E agar plates. The plates were incubated for 12 h and the colonies were counted. Each sample was analyzed in triplicate.

### Statistical analysis

The expression levels of the miRNA and mRNA were calculated using the 2^–ΔΔCT^ method, in which the value represents the n-fold change in the test samples relative to their corresponding control. The data are expressed as the mean ± SD. The statistical significance was subjected to one-way analysis of variance (ANOVA) followed by multiple Duncan tests to determine the differences in the mean values among the controls. A nonparametric test was employed to compare the challenge and control groups at each sampling time. Any significant differences relative to the control for each time point are indicated with an asterisk at *P* < 0.05 and two asterisks at *P* < 0.01.

## Additional Information

**How to cite this article**: Lu, M. *et al*. miRNA-133 augments coelomocyte phagocytosis in bacteria-challenged Apostichopus japonicus via targeting the TLR component of *IRAK-1 in vitro* and *in vivo*. *Sci. Rep*. **5**, 12608; doi: 10.1038/srep12608 (2015).

## Figures and Tables

**Table 1:** The putative miR-133 targets identified by RNA-seq.

miRNA	Putative targets
	U3 small nucleolar ribonucleoprotein
	Metalloproteinase inhibitor 1
	Heat shock protein 67B3
	Neurogenic locus notch homolog protein 1
	Lysozyme 1
	[Cu-Zn] Superoxide dismutase
	Excitatory amino acid transporter
	Methyltransferase-related protein
	Angiotensin-converting enzyme
	Forkhead transcription factor A
miR-133	Nitric oxide synthase
	Zinc transporter ZIP4
	Peptidylglycine alpha-hydroxylating monooxygenase
	Actin-10
	Aldehyde dehydrogenase family 7 member B4
	G protein-coupled receptor 126
	Alkaline serine protease
	Tyrosine-protein phosphatase
	Calumenin-B
	Interleukin-1 receptor-associated kinase 1

**Table 2:** PCR primer and interference sequence information in this study.

Names	Sequences(5′-3′)	Application
Adapter dT	GGCCACGCGTCGATAGTACT17	RACE adapter
IRAK-1-3-1	GTTTGCTCACGGCACTCTCC	3′RACE
IRAK-1-3-2	GCCAGACAGACCGTTCGGAC	
miR-133 mimics		
Sense	UUUGGUCCCCUUCAACCAGCCGU	
Antisense	GGCUGGUUGAAGGGGACCAAAUU	miRNA interference
Negative control		
Sense	UUCUUCGAACGUGUCACGUTT	
Antisense	ACGUGACACGUUCGGAGAATT	
miR-133 inhibitor	ACGGCUGGUUGAAGGGGACCAAA	
Negative control	CAGUACUUUUGUGUAGUACAA	miRNA interference
si-AjIRAK-1(681)		
Sense	GCAGCCAAGCAUGAACUAUTT	
Antisense	AUAGUUCAUGCUUGGCUGCTT	RNA silencing
Negative control		
Sense	UUCUCCGAACGUGUCACGUTT	
Antisense	ACGUGACACGUUCGGAGAATT	
RNU6B	CGTGAAGCGTTCCATATTTTAA	Real-time PCR
	TaKaRa miScript universal primer	
miR-133	TTTGGTCCCCTTCAACCAGCCGT	Real-time PCR
	TaKaRa miScript universal primer	
18s RNA	CGAGTCGTGGGAGATTTTT	Real-time PCR
	CACTTTGGCTGCTTTGAAC	
AjIRAK-1	ACCGTCCTTCCGTGGTGGTCATC	Real-time PCR
	CTGCGTCGTAACATCCGTTCTGC	
AjTRAF6	AGGAGCGGGAAAGGAAGCAGA	Real-time PCR
	TAGCCGTAGAGCGCCGTGTAG	
AjP105	TCTTCGCATTCCATTGAGCTG	Real-time PCR
	ATGGTCCTTCACAGCCGTATCT	
AjIκB	ACAGGAGTCGTTTGATGATTGG	Real-time PCR
	GTTTCTTCTTGTGTTTGGCGTTC	
